# Neuromuscular fatigability at high altitude: Lowlanders with acute and chronic exposure, and native highlanders

**DOI:** 10.1111/apha.13788

**Published:** 2022-01-25

**Authors:** Luca Ruggiero, Scott W. D. Harrison, Charles L. Rice, Chris J. McNeil

**Affiliations:** ^1^ 9304 Laboratory of Physiomechanics of Locomotion Department of Pathophysiology and Transplantation University of Milan Milan Italy; ^2^ School of Kinesiology Faculty of Health Sciences The University of Western Ontario London Ontario Canada; ^3^ Department of Anatomy and Cell Biology Schulich School of Medicine and Dentistry The University of Western Ontario London Ontario Canada; ^4^ Centre for Heart, Lung & Vascular Health School of Health and Exercise Sciences University of British Columbia Kelowna British Columbia Canada

**Keywords:** central fatigue, hypoxia, peripheral fatigue, Sherpa, supraspinal fatigue

## Abstract

Ascent to high altitude is accompanied by a reduction in partial pressure of inspired oxygen, which leads to interconnected adjustments within the neuromuscular system. This review describes the unique challenge that such an environment poses to neuromuscular fatigability (peripheral, central and supraspinal) for individuals who normally reside near to sea level (SL) (<1000 m; ie, lowlanders) and for native highlanders, who represent the manifestation of high altitude‐related heritable adaptations across millennia. Firstly, the effect of acute exposure to high altitude‐related hypoxia on neuromuscular fatigability will be examined. Under these conditions, both supraspinal and peripheral fatigability are increased compared with SL. The specific mechanisms contributing to impaired performance are dependent on the exercise paradigm and amount of muscle mass involved. Next, the effect of chronic exposure to high altitude (ie, acclimatization of ~7‐28 days) will be considered. With acclimatization, supraspinal fatigability is restored to SL values, regardless of the amount of muscle mass involved, whereas peripheral fatigability remains greater than SL except when exercise involves a small amount of muscle mass (eg, knee extensors). Indeed, when whole‐body exercise is involved, peripheral fatigability is not different to acute high‐altitude exposure, due to competing positive (haematological and muscle metabolic) and negative (respiratory‐mediated) effects of acclimatization on neuromuscular performance. In the final section, we consider evolutionary adaptations of native highlanders (primarily Himalayans of Tibet and Nepal) that may account for their superior performance at altitude and lesser degree of neuromuscular fatigability compared with acclimatized lowlanders, for both single‐joint and whole‐body exercise.

## 1 INTRODUCTION

Human performance is often limited by fatigue,^1^ which we will define as an exercise‐induced reduction in the ability to exert muscle force or power, regardless of whether the task can still be performed successfully (modified from Ref. [2]). To localize the sites of impairment within the motor pathway, separate terms are used to describe force loss due to mechanisms at or distal to the neuromuscular junction (*peripheral fatigue*), proximal to the neuromuscular junction (*central fatigue*) and at or above the motor cortex (*supraspinal fatigue*).^3^ The reductions of muscle output, and the mechanisms responsible, are dependent on the characteristics of the fatiguing task.^2^ For example, the proportion of force loss due to central fatigue increases with task duration.^4^, ^5^ Besides the influence of the task characteristics, the mechanisms of neuromuscular fatigability can be affected by environmental conditions such as temperature and availability of oxygen.^6^, ^7^ This review will explore the latter condition in the context of high altitude.

The study of neuromuscular function with exposure to high altitude has value for several reasons. Firstly, approximately 81.6 million people live permanently at altitudes ≥2500 m,^8^ and >40 million people transiently go to high altitude for work or leisure every year.^9^ Secondly, understanding the aetiology of neuromuscular fatigability at high altitude may lead to strategies to limit development of fatigue, which could be critical during activities such as rescue operations. Thirdly, just as other experimental paradigms represent analogues for clinical conditions (eg, cold for hypothermia, heat for hyperthermia, bed rest or chronic unloading for prolonged inactivity or microgravity), the study of human responses to high altitude may offer insight into the pathophysiology of critical illnesses accompanied by hypoxaemia.^10^

High altitude (3500‐6250 m) is a unique challenge for the human body. This is particularly true when those who reside below 1000 m (lowlanders) transition swiftly from these sea level (SL) conditions to real or simulated (eg, normobaric or hypobaric hypoxic chambers) high altitude (acute high‐altitude exposure; AH). With AH, rapid adjustments to many physiological systems of the human body are required to maintain homeostasis. For example, to counteract reductions in haemoglobin saturation (S_a_O_2_) and arterial oxygen content (C_a_O_2_) that follow a decline in pressure of inspired oxygen (P_I_O_2_), heart rate (HR) and ventilation (V̇_E_) increase to preserve oxygen delivery (DO_2_) to muscles and organs. From a performance standpoint, the most immediate consequences compared with SL are decreased maximal aerobic power^11^ and increased neuromuscular fatigability.^12^ In contrast, maximal anaerobic power is typically unaffected with AH.^13^

With chronic (real or simulated) high‐altitude exposure (CH), positive (acclimatization; eg, greater V̇_E_ than AH, polycythaemia, ie, augmented red blood cell number) and negative (eg, hypoxic pulmonary vasoconstriction) interconnected physiological adaptations occur.^14^, ^15^ Functionally, maximal aerobic power improves compared with AH, whereas maximal anaerobic power is unchanged.^16^, ^17^ Neuromuscular fatigability can be either ameliorated from AH^18^, ^19^ or unchanged,^20^ depending on the fatiguing task and locus examined; however, with relatively few studies in the area, much remains unknown.

Despite the many positive adaptations, acclimatized lowlanders do not achieve the remarkable capacity of native highlanders (eg, Sherpa of Nepal and Tibetans) for physical exertion in the high‐altitude environment. This is perhaps unsurprising because the astonishing capacity of these peoples resides in their genotypic and phenotypic adaptations over millennia^21^, ^22^, ^23^; eg, Sherpa of Nepal are descendants of people who have inhabited the Tibetan Plateau for >25 000 years.^24^ Seminal studies as well as reviews have been published on the muscle ultrastructure and metabolic features as well as cardiovascular and respiratory physiology of native highlanders^23^, ^25^, ^26^, ^27^, ^28^; however, none have focussed on the beneficial effects that such features yield for neuromuscular fatigability in these populations at high altitude.

The scope of this review is to describe how neuromuscular fatigability in lowlanders is affected with AH (between 3500 and 6250 m) and CH (short‐ to medium‐term high‐altitude exposure, ~7‐28 days), as well as consider how fatigability is influenced by heritable adaptations and lifetime exposure to high altitude in native highlanders. Regarding the effect of high altitude on neuromuscular physiology, numerous resources exist, from sections of the cornerstone book of Ward, Milledge and West,^29^ to excellent reviews on muscle ultrastructure, energetics and contractile characteristics,^16^, ^23^, ^30^, ^31^ cerebral blood flow, cerebral function and exercise tolerance.^32^, ^33^, ^34^ Whereas former reviews collectively characterize the effect of high‐altitude exposure on neuromuscular fatigability, the aspect of task‐specificity has not been explored in depth as it relates to the continuum of acute, chronic and lifetime (ancestral) exposure to high altitude, and the evolutionary adaptations of highlanders to cope with fatigability in such an environment. This review focuses solely on neuromuscular fatigability and provides a comprehensive examination of important factors such as: duration of exposure to high altitude; modality and type of exercise; amount of muscle mass involved; acclimatization in the context of heritable adaptations and a lifetime of high‐altitude exposure (primarily for Himalayan Tibetans and Sherpa). This final point is particularly useful because a comparison of lowlanders to those who represent the gold standard of adaptability to high altitude provides insight into limitations to acclimatization for those who reside near to SL.

## 2 THE CHALLENGE AT HIGH ALTITUDE

From a value of about 760 mm Hg at SL, air pressure (P_b_) decreases with increasing altitude, which leads to decreased pressure of inspired gases. As aerobic organisms depend on O_2_, the decline of P_I_O_2_ with altitude represents a notable stressor for humans. For a latitude between 15 and 45° (and as an average between summer and winter months, with P_b_ greater in summer), P_b_ (in mm Hg) can be determined as:

$$P_{b}=\exp\left[6.63268‐0.1112\times \left(h\right)‐0.00149\times {\left(h\right)}^{2}\right]$$

with *h* representing the elevation in kilometres (relative to SL).^35^ Accounting for the fraction of oxygen in air (F_I_O_2_; 0.2093) and the water vapour pressure (47 mm Hg), P_I_O_2_ (in mm Hg) can be calculated as:

$$P_{I}O_{2}=0.2093\times \left(P_{b}‐47\right)$$

To solve for P_I_O_2_ at a given altitude or elevation, the two equations can be combined:

$$P_{I}O_{2}=‐9.8371+\exp\;\left[5.06869‐0.1112\times \left(h\right)‐0.00149\times {\left(h\right)}^{2}\right]$$

**FIGURE 1:**
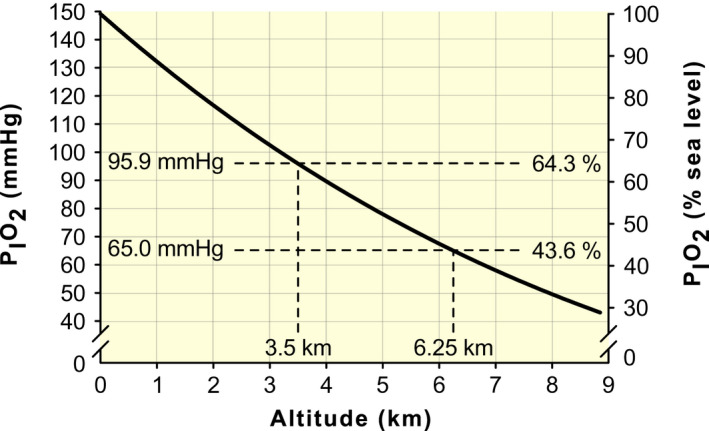
Pressure of inspired oxygen (P_I_O_2_) as a function of altitude. P_I_O_2_ (mm Hg) was calculated as −9.8371 + exp [5.06869−0.1112 × (*h*)−0.00149 × (*h*)^2^], with *h* representing the elevation in kilometres (relative to sea level; see text for details). At 3.5 and 6.25 km, the lower and upper limits of high altitude in the present review, P_I_O_2_ is ~95.9 and 65.0 mm Hg, respectively, ie, 64.3% and 43.6% of P_I_O_2_ at sea level (149 mm Hg)

It follows that the P_I_O_2_ at 3500 and 6250 (lower and upper limits of high altitude in the present review) is 64.3% and 43.6% of P_I_O_2_ at SL (149 mm Hg), respectively (Figure 1), and at about 5300 m P_I_O_2_ is halved relative to SL. Two other equations can further characterize the challenge of exposure to high altitude in the context of neuromuscular fatigability:

$$C_{a}O_{2}=\left(1.36\times \left[\text{Hb}\right]\times S_{a}O_{2}\right)+\left(0.003\times P_{a}O_{2}\right)$$

$${\text{DO}}_{2}=C_{a}O_{2}\times \left(\text{BF}/100\right)$$

with C_a_O_2_ in mL/dL, [Hb] (concentration of haemoglobin) in g/dL, S_a_O_2_ in % (NB: if estimated with pulse oximetry, it is referred to as S_p_O_2_), 1.36 the affinity of oxygen for haemoglobin, P_a_O_2_ (partial pressure of arterial oxygen) in mm Hg, 0.003 the solubility of O_2_ in the plasma, and BF (blood flow) as well as DO_2_ in mL/min.

Immediate ascent to high altitude leads to a decline in P_I_O_2_, and if all else remains equal, to reduced P_a_O_2_, S_a_O_2_, C_a_O_2_ and DO_2_. In this acute situation, compensatory adjustments attempt to mitigate these declines, chiefly by increased V̇_E_.^36^ Despite this, the O_2_ pressure gradients at each step of the O_2_ cascade, from ambient air to human muscle, are reduced with acute exposure to P_I_O_2_ encountered at high altitude.^37^, ^38^ These reductions do not present limitations for O_2_ uptake in the resting skeletal muscle.^39^ However, as muscle oxygen consumption (V̇O_2_) is largely determined by DO_2_ as well as the gradient between capillary and muscle intracellular PO_2_, and an increased DO_2_ does not offset the decline in this gradient, muscle V̇O_2_ at submaximal absolute workloads and maximal V̇O_2_ (V̇O_2max_) are lower with high altitude‐related hypoxia.^40^, ^41^

With short‐ to medium‐term CH (~7‐28 days of exposure to high altitude), despite acclimatization, restoration of P_a_O_2_ and performance to SL never occur.^14^ As with AH, the most important physiological response with acclimatization is hyperventilation.^14^ That is, V̇_E_ (as well as the hypoxic ventilatory response) increases gradually over 1‐2 weeks at altitude.^42^, ^43^, ^44^ This increased ventilation is especially important for the early adaptations with CH, as it contributes to the increase of S_a_O_2_ and P_a_O_2_ by mitigating the reduction in alveolar PO_2_ at altitude, and by increasing [Hb] through respiratory alkalosis‐induced diuresis.^17^, ^42^ Indeed, with CH, acclimatized individuals typically have greater P_a_O_2_ and lower P_a_CO_2_ than unacclimatized individuals at the same altitude.^45^ The second most important response in acclimatization is increased [Hb].^14^ Initially (up to 2 weeks), reduced plasma volume is the main determinant of increased [Hb], whereas later (>2 weeks), polycythaemia prevails.^46^, ^47^ After ~1 week at high altitude, increased [Hb], rather than P_a_O_2_, is the main contributor to the partial recovery of C_a_O_2_ to values at SL^46^ (see also figure 2 in Ref. [45]).

In addition to the aforementioned systemic responses with CH that collectively improve P_a_O_2_, S_a_O_2_, [Hb] and C_a_O_2_, acclimatization occurs also in the muscle, at the ultrastructural and metabolic levels.^23^, ^30^, ^48^ Although such acclimatization responses generally induce a reduction in peripheral fatigability with CH compared with AH, one caveat with acclimatization remains, ie, the increased work of breathing because of ventilatory acclimatization.^49^, ^50^ Further details and the implications of this will be presented in the section regarding neuromuscular fatigability with CH.

## 3 EXERCISE CONSIDERATIONS TO STUDY NEUROMUSCULAR FATIGABILITY AT HIGH ALTITUDE

Before delving into the effect of exposure to high altitude on neuromuscular fatigability, it is important to present a few considerations related to task specificity, in particular the amount of muscle mass involved, the determination of the targeted intensity and the exercise paradigm.

### 3.1 Amount of muscle mass involved

**FIGURE 2:**
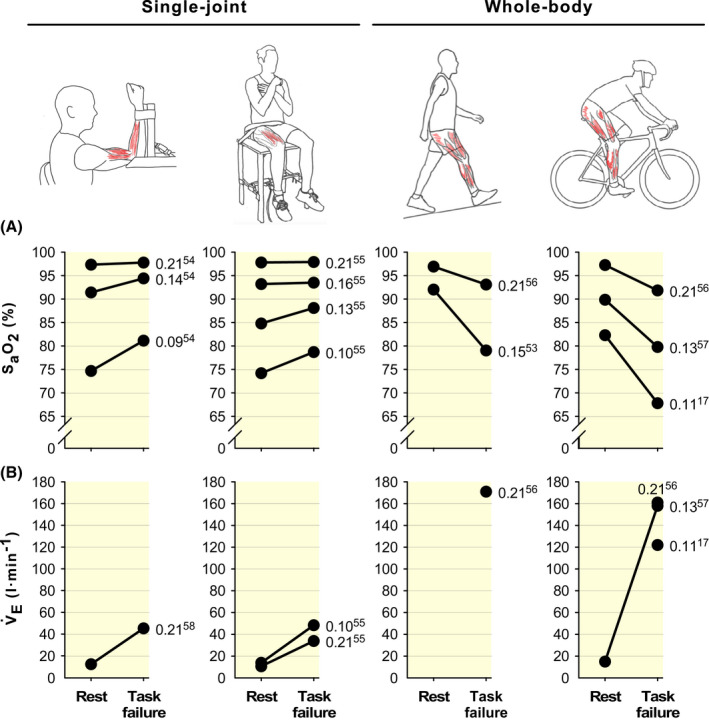
Arterial oxygen saturation (S_a_O_2_) and ventilation (V̇_E_) in single‐joint and whole‐body exercise at rest and at task failure, with different inspired fractions of oxygen (F_I_O_2_). A, exercise‐induced arterial hypoxaemia (indicated by a decrease in S_a_O_2_ with exercise, consequent to declined P_a_O_2_) occurs with whole‐body but not with single‐joint tasks. B, greater V̇_E_ in whole‐body than single‐joint exercise, and as a consequence work of breathing, is associated with greater respiratory muscle metaboreflex (see text for details). All data have been retrieved from figures (using WebPlotDigitizer v4.3) or tables of original articles. Numbers around data points indicate the F_I_O_2_ used in the study, with the reference number considered for data in superscript. A F_I_O_2_ value of 0.21 corresponds to sea level, whereas F_I_O_2_ values of 0.16, 0.15, 0.14, 0.13, 0.11, 0.10 and 0.09 correspond to simulated altitude‐related hypoxia at ~2200, 2700, 3300, 3800, 5000, 5600, 6300 m

The amount of muscle mass involved markedly influences the aetiology of neuromuscular fatigability. This is a well‐known factor for exercise with AH and CH,^41^, ^51^ a feature also recently highlighted in the context of neuromuscular fatigability with normoxic exercise.^52^ When evaluating neuromuscular fatigability with high‐intensity whole‐body exercise with AH or CH, it is necessary to consider potential cardiorespiratory limitations and associated hypoxic‐related impairments. Firstly, exercise‐induced arterial hypoxaemia (a decline in P_a_O_2_ during exercise)^53^ develops with AH and CH with whole‐body but not with single‐joint exercise (Figure 2A)^54^, ^55^, ^56^, ^57^, ^58^, which accelerates the rate of peripheral fatigability of the motor muscles.^59^ Secondly, in the face of a finite cardiac output, greater work of breathing with whole‐body relative to single‐joint exercise (see values of V̇_E_ as an approximate in Figure 2B), and the associated respiratory muscle metaboreflex,^60^ will cause blood flow to be redistributed away from the locomotor muscles,^60^, ^61^ thus exacerbating their fatigability.^62^, ^63^ Considering also that fatigability of respiratory muscles is worsened with severe hypoxia,^64^ the respiratory muscle metaboreflex with AH and CH is further exacerbated compared with SL.^20^, ^65^ As neither exercise‐induced arterial hypoxaemia nor increased work of breathing^58^ are relevant when small muscle mass is involved (see Figure 2), single‐joint exercise is an excellent paradigm to investigate the direct effects of acute or sustained low P_a_O_2_ on the fatigability of the neuromuscular system. However, whole‐body exercise represents an ideal paradigm to study how the cardiorespiratory and neuromuscular systems integrate and adapt in response to the hypoxic challenge.

### 3.2 Determination of the targeted intensity

**FIGURE 3:**
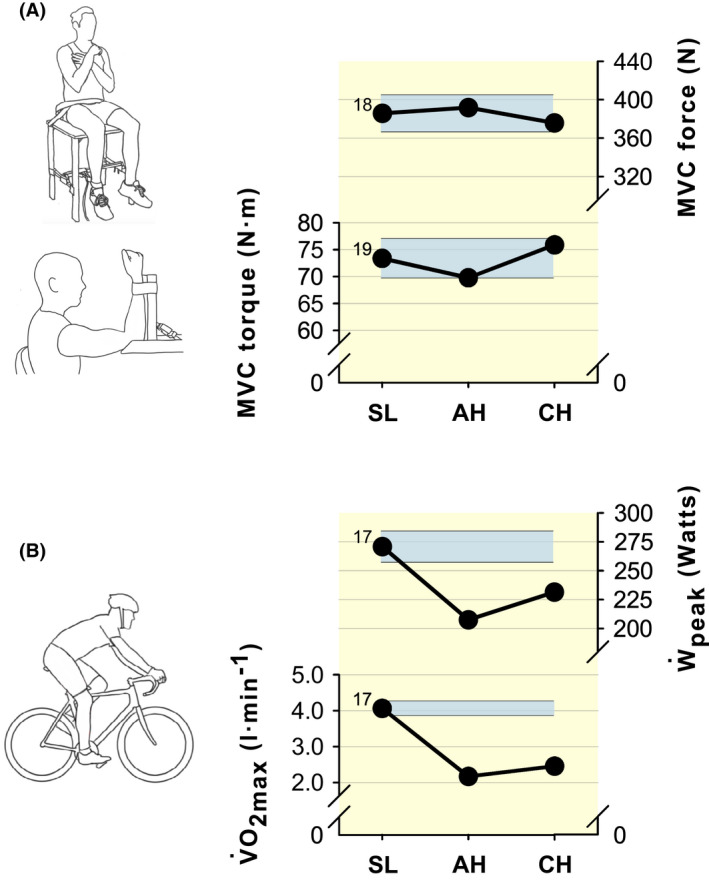
Maximal voluntary contraction (MVC) force or torque, maximal oxygen consumption (V̇O_2max_) and peak power output (Ẇ_peak_) at sea level (SL), and with acute (AH) and chronic (CH) exposure to high altitude. All data have been retrieved from figures (using WebPlotDigitizer v4.3) or tables of original articles corresponding to the reference numbers in superscript. For reference, the turquoise shaded areas indicate the interval between 95% and 105% of values at SL for MVC force, torque, V̇O_2max_ and Ẇ_peak_. A, single‐joint exercise. B, whole‐body exercise (cycle ergometry). For single‐joint exercise, MVC force or torque is typically not affected with AH and CH; it follows that neuromuscular fatigability studies involving single‐joint isometric exercise, using the same relative exercise intensity (% MVC force) across SL, AH and CH, will most likely target the same absolute force or torque. In contrast, as V̇O_2max_ and Ẇ_peak_ are greatly reduced with AH and CH compared with SL, a relative intensity for whole‐body exercise (% V̇O_2max_ or Ẇ_peak_) likely will result in different absolute exercise intensities (work rates) across conditions

For single‐joint exercise, the isometric or isokinetic maximal voluntary contraction (MVC) force of a muscle or a muscle group is typically not affected with AH (Figure 3A).^54^, ^55^, ^66^, ^67^, ^68^ However, there are some reports of a lower MVC force with AH compared with SL.^31^, ^69^ Lower maximal strength may reflect poorer cortical voluntary activation with severe acute hypoxia,^19^, ^70^ owing to an abrupt reduction in cerebral mitochondrial O_2_ pressure.^71^ Similar to AH, with CH (at the same or gradually increasing level of high altitude over days), single‐joint MVC force is typically not different from SL (Figure 3A).^18^, ^19^, ^20^, ^72^, ^73^, ^74^, ^75^ An exception to this would be cases when there is considerable high altitude‐related muscle wasting.^76^, ^77^ However, studies have shown that this muscular atrophy can be greatly limited by increased caloric intake,^78^, ^79^ which would prevent an atrophy‐associated decline in MVC force with CH. It follows that neuromuscular fatigability studies involving single‐joint isometric exercise, using the same relative exercise intensity (% MVC force) across SL, AH and CH, will most likely target the same absolute force (or joint torque). In contrast, a relative intensity for whole‐body exercise (% V̇O_2max_ or peak power output; Ẇ_peak_) likely will result in different absolute exercise intensities (work rates) across conditions because V̇O_2max_ and Ẇ_peak_ are greatly reduced with AH compared with SL, and reductions are still evident with CH (Figure 3B).^16^, ^17^, ^41^

### 3.3 Exercise paradigm

**FIGURE 4:**
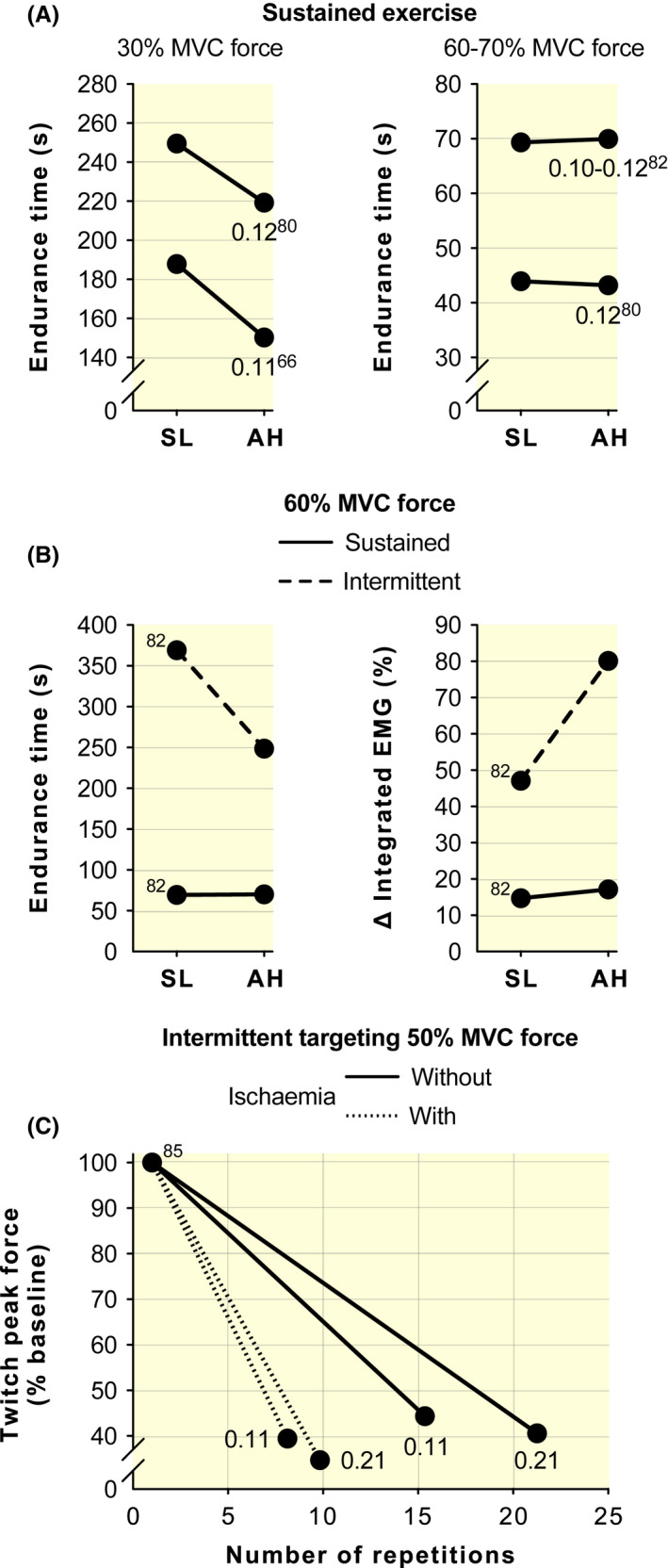
Effect of different single‐joint exercise paradigms on endurance time and peripheral fatigability at sea level (SL) and with acute exposure to high altitude (AH). All data have been retrieved from figures (using WebPlotDigitizer v4.3) or tables of original articles. All plots reflect isometric knee extension exercise until exhaustion, sustained at 30% or 60%‐70% of the maximal voluntary contraction (MVC) force, or intermittent targeting of 50% or 60% MVC force. Numbers around data points indicate the F_I_O_2_ used in the studies considered, with the reference number from which data have been retrieved in superscript. A F_I_O_2_ value of 0.21 corresponds to SL, whereas F_I_O_2_ values between 0.10 and 0.12 correspond to simulated altitude‐related hypoxia between ~4300 and 5600 m. A, sustained contractions have a briefer endurance time with AH compared with SL at 30% MVC force (on the left) but not at 60%‐70% MVC force (on the right). B, endurance time was shorter and peripheral fatigability (estimated indirectly with increased electromyographic activity; EMG) was greater with AH than SL for an intermittent protocol at ~60% MVC force (dashed line), whereas both measures were similar between conditions when the exercise was sustained (continuous line) at the same target intensity. C, the rate of peripheral fatigability was greater with AH than SL with intermittent contractions targeting 50% MVC force (continuous line), but not when the same exercise was repeated in both conditions with ischaemia of the exercising leg (dotted line)

Key considerations for a single‐joint exercise paradigm are the percentage of MVC force targeted and pattern of activation (ie, sustained vs intermittent contractions). Although direct investigations are limited in the context of hypoxia, sustained contractions have a briefer endurance time with AH compared with normoxia (SL) at low intensity (eg, 30% MVC force)^66^, ^80^ but not at high intensity (eg, 60% or 70% MVC force; Figure 4A).^80^, ^81^, ^82^ Studies by Katayama and colleagues^68^, ^82^ demonstrated the importance of contraction type as, in contrast to the findings with a sustained contraction, endurance time was shorter and peripheral fatigability (estimated indirectly with increased electromyographic activity) was greater with AH than SL for an intermittent protocol at 60% MVC force, whereas both measures were similar between conditions when the exercise was sustained at the same target intensity (Figure 4B). Muscle blood flow occlusion can occur with a sustained high‐intensity contraction, and the percentage of MVC force needed to occlude blood flow to muscle fibres is lower for strong than weak individuals.^83^ With a substantial reduction of DO_2_, the accumulation of metabolites as well as their clearance, which are chief determinants of peripheral fatigability,^84^ may be unaffected by reduced initial levels of P_a_O_2_, S_a_O_2_ and C_a_O_2_, ie, AH. Indeed, the rate of peripheral fatigability was greater with AH than SL with intermittent isometric knee extensions targeting 50% MVC force, but not when the exercise was repeated in both conditions with ischaemia of the exercising leg (Figure 4C).^85^ Additionally, muscle oxygenation, as measured by near‐infrared spectroscopy, was not different between AH and SL for sustained high‐intensity single‐joint exercise but lower with AH than SL when contractions were intermittent.^82^ Collectively, these observations indicate that the best insight into the effects of AH and CH on neuromuscular fatigability during single‐joint exercise will be achieved with intermittent contractions or a low‐intensity sustained contraction (ie, <60% MVC force).

With whole‐body exercise, the task paradigms most often involve a constant work rate for a specified time, incremental or constant work rate to exhaustion, or time trials (maximal distance in a specified time, ie, work rate is the dependent variable). It is imperative to consider the paradigm chosen when interpreting the effect of AH or CH on neuromuscular fatigability. For example, with a constant absolute work rate for a specified time, both peripheral^86^ and supraspinal^57^ fatigability are greater with AH compared with SL. In contrast, if such a work rate is held to exhaustion, peripheral fatigability is lower,^86^ whereas supraspinal fatigability is greater^57^ with AH compared with SL. The latter is also the case with incremental exercise to exhaustion with AH, when task termination occurs at a lower Ẇ_peak_ with both AH and CH compared with SL.^16^, ^17^ If, however, whole‐body exercise is conducted at a relatively low intensity and for a prolonged time (≥90 min), the same magnitudes of peripheral and supraspinal fatigability are present for SL and AH.^87^, ^88^ Details (and the reasonings) will be reported in the sections specific to neuromuscular fatigability with AH and CH.

## 4 NEUROMUSCULAR FATIGABILITY WITH ACUTE EXPOSURE TO HIGH ALTITUDE

**FIGURE 5:**
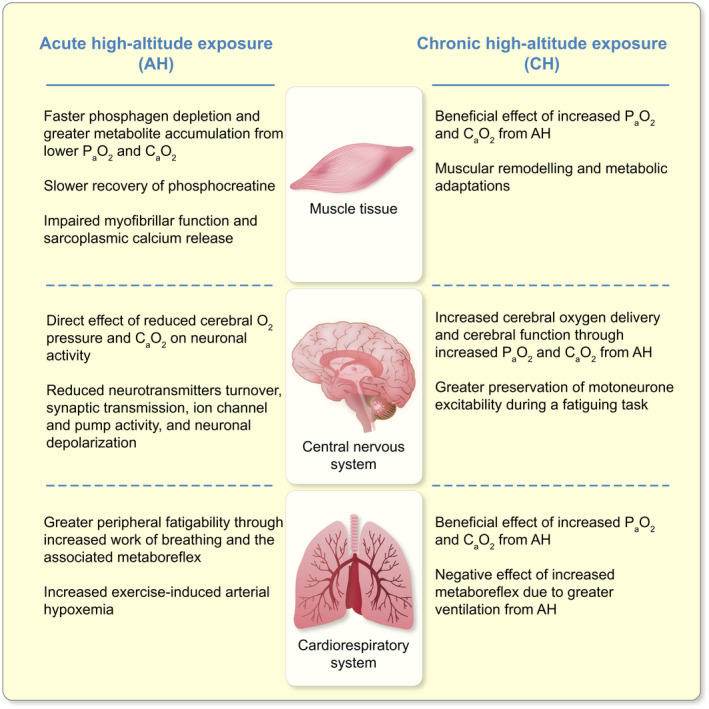
Schematic representation of the mechanisms in the muscle tissue, central nervous system, and cardiorespiratory system, contributing to peripheral and supraspinal fatigability in single‐joint and whole‐body exercise with acute (AH) and chronic (CH) exposure to high altitude. P_a_O_2_, partial pressure of arterial oxygen; C_a_O_2_, arterial oxygen content. The additional influence of the cardiorespiratory system occurs when whole‐body exercise, as opposed to single‐joint, is performed

The current consensus of peripheral and supraspinal fatigability with AH relative to SL for different exercise types is summarized in Table 1. The mechanisms contributing to peripheral and supraspinal fatigability in single‐joint and whole‐body exercise with AH relative to SL are schematically reported in Figure 5.

**TABLE 1:** Peripheral and supraspinal fatigability with acute exposure to high altitude, relative to values at sea level, based on the exercise paradigm

Acute high‐altitude exposure (AH) versus sea level (SL)				
	Single‐joint		Whole‐body	
	PF	SF	PF	SF
Isotime				
Absolute target intensity (force or power)	+	+	+	+
			18, 20, 57, 90, 91	18, 57
Relative target intensity (% MVC force or % Ẇ_peak_)	+	+	=	=
	68, 75, 85	19	88	88
Isointensity				
Exhaustion	−	+	−	+
	55	55, 89	86	57

Note

The plus (+), minus (−) and equal (=) signs indicate that the fatigability measure with AH is greater, lower or not different relative to SL, respectively. Numbers below signs indicate the cited references that support each conclusion. Of note, no studies have been conducted using gold‐standard measures of fatigability for single‐joint exercise at absolute target intensity because MVC force is typically not lower with AH relative to SL (see ‘Determination of the targeted intensity’ subsection in text), which means findings for this situation would mirror those at a relative target intensity.

Abbreviations: MVC, maximal voluntary contraction, PF, peripheral fatigability; SF, supraspinal fatigability; Ẇ_peak_, peak power output.

### 4.1 Single‐joint exercise studies

**FIGURE 6:**
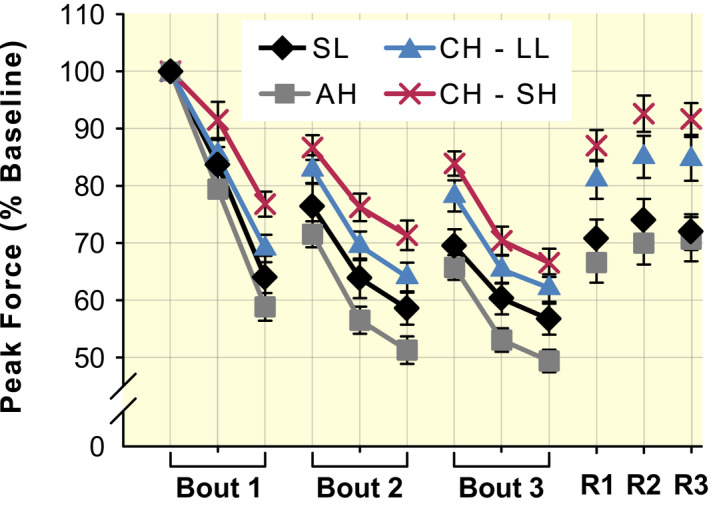
Peak force during and after a unilateral, fatiguing protocol comprised of intermittent electrically evoked contractions of the knee extensors. Data are mean values (±SEM) collected at sea level (SL, black diamonds; n = 11), with acute (AH, grey squares; n = 11) and chronic (CH—LL, blue triangles; n = 11) high‐altitude exposure in lowlanders (data adapted from Ref. [75]), and collected at high altitude from Sherpa (CH—SH, red crosses; n = 10; data adapted from Ref. [150]). Values are reported as percentage of the baseline peak force. The fatiguing task involved three bouts of 75 electrically evoked contractions. For each session, the initial target was 30% of the maximal voluntary contraction force attained that day. Each stimulus train consisted of 12 pulses at 15 Hz (800 ms long), with 800 ms of rest between trains (there were 15 s of rest between bouts of contractions). Data points during the fatiguing protocol represent the mean peak force of the first, middle and last five contractions of each bout. R1, R2 and R3 represent the peak force of single contractions evoked 1, 2 and 3 min after the end of the fatiguing task

Unless the exercise task is a high‐intensity (eg, ≥60% MVC force) sustained contraction (see subsection ‘Exercise paradigm’ of the previous section), the rate of peripheral fatigability is increased with AH compared with SL. This has been demonstrated with voluntary exercise^55^, ^68^, ^85^ as well as intermittent electrically evoked contractions.^75^ As there is no evidence that Na^+^‐K^+^‐ATPase activity^92^ or neuromuscular propagation^19^, ^65^, ^70^, ^93^, ^94^ are impaired with AH relative to SL, excitation–contraction coupling is the most likely explanation for greater peripheral fatigability in the former condition. With AH, the muscular intracellular partial pressure of O_2_ is lower,^39^ which leads to a faster rate of phosphagen depletion and metabolite accumulation^95^, ^96^ and a slower recovery of phosphocreatine.^97^ The direct effect of reduced intracellular O_2_ pressure,^98^ as well as the prominent effects of greater metabolite accumulation (particularly inorganic phosphate) on myofibrillar function and sarcoplasmic Ca^2+^ release^84^, ^99^ would accelerate the decline in muscle tissue contractility with AH relative to SL. This is represented in Figure 6, which shows that the decline of force with AH was greater than at SL for intermittent electrically evoked contractions of the knee extensors (from Ref. [75]).

With AH, relative to SL, the rate of supraspinal fatigability is also accelerated. This has been reported for both lower limb^55^, ^89^ and upper limb muscles.^19^ Of note, when examining central fatigability as opposed to supraspinal fatigability (ie, stimulation is applied to the peripheral nerve rather than motor cortex), no differences have typically been reported between SL and AH.^68^, ^100^ For single joint exercise, the greater supraspinal fatigability with AH relative to SL may be due to a direct effect of reduced cerebral O_2_ pressure on neuronal activity.^101^ Indeed, reduced cerebral O_2_ pressure with AH markedly decreases electroencephalographic activity and complexity of the signal (reversible after reoxygenation),^102^, ^103^, ^104^ the turnover of neurotransmitters,^105^ synaptic transmission, ion channel and pump activity, and neuronal depolarization (for review see Refs [32, 33, 34] and [106]). Alternatively, greater supraspinal fatigability with AH relative to SL could relate to increased group III/IV muscle afferent feedback, either from increased resting discharge rate^107^, ^108^ or from accelerated formation of metabolites during the fatiguing task.^96^ When using vascular occlusion (ischaemia) of the exercising elbow flexors to ensure a similar muscular milieu between conditions, task failure occurred sooner with AH than SL, and cerebral oxygenation decreased in the simulated AH condition only.^54^ This suggests that reduced cerebral oxygenation, independent of afferent feedback from the working muscles, reduced exercise performance.^54^ Support for this suggestion comes from a study by Calbet and colleagues^41^ who reported administration of normoxic or hyperoxic gas at the point of exhaustion during an incremental concentric knee extension task with AH enabled participants to continue exercising. Furthermore, when cerebral oxygenation with AH was increased by supplementing inspiratory CO_2_, supraspinal fatigability was reduced at the expense of greater peripheral fatigability (due to greater respiratory‐induced acidosis),^89^ with no net effects on performance. Collectively, such findings indicate that cerebral O_2_ pressure as well as cerebral oxygenation contribute substantially to the exacerbation of supraspinal fatigability with AH.

### 4.2 Whole‐body exercise studies

Unlike single‐joint exercise, whole‐body exercise is influenced by cardiorespiratory limitations and any associated hypoxic‐related impairments (chiefly exercise‐induced arterial hypoxaemia and the respiratory muscle metaboreflex; see subsection ‘Amount of muscle mass involved’ of the previous section). However, the effects of AH on peripheral and supraspinal fatigability during whole‐body exercise corroborate findings from single‐joint studies with AH.

When exercising at the same absolute intensity for the same duration (except for a prolonged time, ie, ≥90 min, see paragraph at the end of this section), peripheral fatigability is increased with AH compared to SL,^18^, ^20^, ^57^, ^90^, ^91^ presumably due in part to greater metabolite accumulation with AH.^109^ Additionally, increased exercise‐induced arterial hypoxaemia and work of breathing with AH relative to SL can exacerbate the development of peripheral fatigability in the former condition.^53^, ^59^, ^62^, ^63^

With whole‐body exercise at the same absolute intensity at SL and with AH, the rate of supraspinal fatigability is also accelerated.^18^, ^57^ When epidural anesthesia was used to block group III/IV afferent feedback from the working muscles during cycling with AH, time to exhaustion was not different from the trial with intact feedback.^110^ Additionally, when hyperoxic gas was delivered at the point of exhaustion during cycling with AH, EMG activity of locomotor muscles as well as cerebral oxygenation were rapidly increased, and exercise continued.^51^, ^86^, ^111^ These findings indicate indirectly that the performance reduction with AH relative to SL has a central component independent of afferent feedback, and sensitive to severely declined P_a_O_2_, which may be responsible for greater supraspinal fatigability in the former condition.^34^, ^106^

A hallmark of whole‐body exercise with AH is greater V̇_E_ compared with SL. Although this is a necessary cardiorespiratory adjustment to mitigate the decline in P_a_O_2_ with severe hypoxia, two inevitable drawbacks are increased work of breathing and hyperventilation‐induced hypocapnia (ie, decreased partial pressure of arterial CO_2_, P_a_CO_2_). As explained previously, increased work of breathing with AH relative to SL increases the rate of peripheral fatigability in locomotor muscles, independent of hypoxia.^62^, ^65^ Hypocapnia has a profound effect on supraspinal centres, leading to cerebral vasoconstriction and decreased cerebral blood flow.^112^ Indeed, decreased P_a_CO_2_ during exercise is concomitant to reduced cerebral blood flow and cerebral oxygenation,^113^, ^114^ which may facilitate supraspinal fatigability.^57^ If the decline of P_a_CO_2_ during whole‐body exercise is prevented (isocapnia) by breathing CO_2_‐enriched air, cerebral blood flow and oxygen delivery are improved, with no effects on performance.^115^, ^116^, ^117^ Although these studies did not measure peripheral, central or supraspinal fatigability, evidence from the work of Rupp and colleagues^89^ (single‐joint exercise) indicates that, in the presence of CO_2_ clamping, the contribution of peripheral fatigability increases, whereas supraspinal fatigability decreases.

Even though AH accelerates the rate of both peripheral and supraspinal fatigability, if single‐joint or whole‐body exercises are conducted to exhaustion (at the same absolute intensity), peripheral fatigability will be lower^55^, ^86^ but supraspinal fatigability will be greater^55^, ^57^, ^89^ at the end of the exercise with AH compared with SL. That is, due to the high sensitivity of supraspinal centres to reduced level of cerebral O_2_ pressure and cerebral oxygenation with AH, the major determinant of exercise performance switches from a predominantly peripheral origin to a hypoxia‐sensitive supraspinal component of fatigability.^57^, ^86^, ^118^

Interestingly, when whole‐body exercise is performed for a prolonged duration and at a relatively low exercise intensity (90 min at a work rate corresponding to ~50% V̇O_2max_ at SL in Ref. [87]; 3 bouts of 80 min at ~45% Ẇ_peak_ for SL and AH in Ref. [88]), comparable magnitudes of peripheral fatigability,^87^, ^88^ supraspinal fatigability and ratings of perceived effort^88^ are present at the end of the exercise for AH and SL.

Overall (see Table 1 for a schematic summary), the evidence presented above indicates that with AH, when targeting the same absolute intensity as at SL for a specified time (eg, absolute force or power), both peripheral and supraspinal fatigability are worsened with AH for both single‐joint and whole‐body exercise. For single‐joint exercise only, if a relative intensity (% MVC force or torque) is targeted for a given period of time, both peripheral and supraspinal fatigability are greater with AH compared with SL. In contrast, for whole‐body exercise, if a relative exercise intensity (eg, % Ẇ_peak_) is performed for the same duration of time, peripheral and supraspinal fatigability are comparable between conditions. Finally, if single‐joint or whole‐body exercise is held to exhaustion, peripheral fatigability is lower, whereas supraspinal fatigability is higher for AH relative to SL.

## 5 NEUROMUSCULAR FATIGABILITY WITH CHRONIC EXPOSURE TO HIGH ALTITUDE

Based on the balance of data from existing studies, peripheral and supraspinal fatigability with CH relative to SL are summarized for different exercise types in Table 2. Figure 5 provides a schematic of the mechanisms contributing to peripheral and supraspinal fatigability in single‐joint and whole‐body exercise with CH relative to SL.

**TABLE 2:** Peripheral and supraspinal fatigability with chronic exposure to high altitude, relative to values at sea level, based on the exercise paradigm

Chronic high‐altitude exposure (CH) versus sea level (SL)				
	Single‐joint		Whole‐body	
	PF	SF	PF	SF
Isotime				
Absolute target intensity (force or power)	=	=	+	=
			18, 20	18
Relative target intensity (% MVC force or % Ẇ_peak_)	=	=	?	?
	75	19		
Isointensity				
Exhaustion	?	?	?	?

Note

The plus (+) and equal (=) signs indicate that the fatigability measure with CH is greater or not different relative to SL, respectively. Numbers below signs indicate the cited references that support each conclusion. Question marks indicate that no studies have been conducted that use gold‐standard measurements to probe neuromuscular fatigability. Of note, no studies have been conducted using gold‐standard measures of fatigability for single‐joint exercise at absolute target intensity because MVC force is typically not lower with CH relative to SL (see ‘Determination of the targeted intensity’ subsection in text), which means findings for this situation would mirror those at a relative target intensity.

Abbreviations: MVC, maximal voluntary contraction, Ẇ_peak_, peak power output; PF, peripheral fatigability; SF, supraspinal fatigability.

### 5.1 Single‐joint exercise studies

When measures such as the decline in MVC force or changes in the root mean square or integrated surface EMG are used to infer the effect of CH on neuromuscular fatigability, findings are equivocal. That is, some studies report that values with CH remain impaired relative to SL,^74^, ^119^ whereas others report no difference between CH and SL data.^51^, ^120^, ^121^ When more detailed measures are taken (ie, with the use of external stimulation), peripheral, central and supraspinal fatigability with CH are restored to SL values.^19^, ^75^ The seminal study of Garner and colleagues^72^ reported that with CH, peripheral fatigability was greater relative to SL. However, measures were taken at extreme altitudes (ie, ~6750 and 8050 m) after 24 and 35 days of simulated hypobaric hypoxia, with a lack of uniform altitude in the days before testing (progressively decreasing levels of P_I_O_2_). Thus, it is hard to interpret such measures as representative of the adaptations of neuromuscular fatigability with CH.

With CH, findings regarding neuromuscular propagation (estimated as the peak‐to‐peak amplitude of the maximal compound muscle action potential; M‐wave) are equivocal, with reports that values with CH are lower,^19^, ^122^ not different^20^ or higher^18^ than SL. A decline in the M‐wave amplitude with CH may be due to decreased concentration of skeletal muscle Na^+^‐K^+^‐ATPase,^123^ which would be a favourable adaptation to severe hypoxia in lowlanders as it would reduce ATP demand.^124^ Indeed, with temporary reoxygenation at high altitude (P_I_O_2_ = 140 mm Hg), impairments in the amplitude and duration of the maximal compound muscle action potential were not resolved,^122^ corroborating the structural (decreased Na^+^‐K^+^‐ATPase concentration) rather than transient nature of such changes.

Despite the possibility that neuromuscular propagation may be impaired with CH, peripheral fatigability (peak force and contractile impulse during an electrically evoked fatiguing protocol of the knee‐extensors) for isotime exercise was lower than AH, and restored to SL values.^75^ One reason for such restoration is the sizeable increase of P_a_O_2_ and C_a_O_2_ with CH relative to AH.^46^ Additionally, with CH, muscular remodelling and metabolic adaptations occur, which are dependent on the magnitude of altitude‐related hypoxia and the duration of exposure.^48^ For the altitude and duration considered in the present review (ie, 3500‐6250 m, and ~7‐28 days), changes in muscle fibre type are not expected,^125^ whereas reductions in myofibrillar proteins or the cross‐sectional area of whole muscle and single fibres may occur.^125^, ^126^, ^127^ Although periods at high altitude longer than 28 days are typically required for mitochondrial density to decrease,^48^, ^128^, ^129^ mitochondria‐specific enzymes related to β‐oxidation, the tricarboxylic acid cycle and oxidative phosphorylation are reduced already between 7 and 28 days.^28^, ^48^, ^130^, ^131^, ^132^ In other adaptations, the control between ATP supply and demand is greater^28^, ^133^ and, due to a shift towards greater dependency on glucose oxidation and improved mitochondrial coupling efficiency for oxygen phosphorylation, so is oxygen efficiency.^38^, ^48^, ^131^, ^134^ These muscular metabolic changes with CH may also explain the faster recovery of muscle force (despite the same end‐exercise peripheral fatigability) after intermittent electrically evoked contractions compared with SL (see figure 2 in Ref. [75]).

With CH, supraspinal fatigability for single‐joint exercise is restored to SL values.^19^ The most likely reason for the improvement from AH to CH is the considerable increase of P_a_O_2_ (and, in turn, cerebral O_2_ pressure) and C_a_O_2_, with acclimatization to high altitude. Indeed, NIRS‐related indices of cerebral oxygenation during single‐joint exercise are similar to those at SL,^19^ indicating that the balance between O_2_ supply and consumption^135^ is not different between the two conditions. Another factor that might favour restoration of supraspinal fatigability with CH to SL values is preservation of motoneurone excitability during a fatiguing task.^19^ That is, unlike the fatigue‐related reduction of motoneurone excitability identified for both AH and SL, the cervicomedullary motor evoked potential did not decrease when an intermittent isometric task was performed with CH.^19^ Of note, the cervicomedullary motor evoked potential was recorded during the silent period induced by transcranial magnetic stimulation, which eliminates the confound of unknown descending drive on this measure.^136^, ^137^ Preservation of motoneurone excitability with CH may be due to increased sympathetic nervous system activity^138^, ^139^ that leads to greater circulating epinephrine and norepinephrine concentrations.^79^, ^140^, ^141^ More excitable motoneurones would require less descending drive for a given muscle output, which would mitigate the functional consequence of any impairment to cortical drive.

### 5.2 Whole‐body exercise studies

A plethora of research has investigated the effect of acclimatization to high altitude on whole‐body exercise performance, particularly using cycling as the exercising task. Most commonly, incremental (eg, Refs [41] and [142]) or isointensity (same absolute work rate) exercise until exhaustion (eg, Refs [51] and [143]) has been used to determine measures such as Ẇ_peak_, V̇O_2max_, concentration of blood lactate (to study the presence or absence of the lactate paradox in lowlanders; eg, Refs [141, 143, 144]), EMG activity or time to exhaustion. However, to our knowledge, only two studies^18^, ^20^ have evaluated neuromuscular fatigability with CH using external stimulation to probe peripheral, central and/or supraspinal fatigability.

Differently from single‐joint exercise, when peripheral fatigability with CH is ameliorated from AH and recovers to values at SL, peripheral fatigability of quadriceps femoris following whole‐body exercise is not different between CH and AH, with both conditions showing greater impairment of the potentiated resting twitch force compared with SL.^18^, ^20^ This absence of an improvement from AH to CH could reflect the interplay of adaptations with CH that would positively (increased P_a_O_2_ and C_a_O_2_, as well as metabolic muscular adaptations) or negatively (greater metaboreflex‐mediated shunting of blood away from the working locomotor muscles due to greater V̇_E_) influence peripheral fatigability. Each of these mechanisms is described in more detail in the preceding sections. The interplay of these mechanisms with CH should be investigated further, particularly with superior measures of peripheral fatigability (see ‘Additional Considerations’ section).

In terms of supraspinal and central fatigability, similar to the findings with single‐joint exercise, acclimatization leads to an amelioration of the impairments seen with AH.^18^, ^20^ This may be due to increased P_a_O_2_ and C_a_O_2_, as well as estimated cerebral DO_2_ during exercise, from AH to CH.^18^ Overall (see Table 2 for a schematic summary), based on the balance of data from existing studies, when single‐joint exercise involves the same absolute or relative (ie, % MVC) force or torque for the same period of time, both peripheral and supraspinal fatigability are not different between CH and SL. Differently, for whole‐body exercise, if the same absolute intensity (work rate) is targeted, peripheral fatigue is worsened with CH relative to SL, whereas supraspinal fatigue is unchanged. No studies have used gold‐standard measurements to compare peripheral and supraspinal fatigability between CH and SL for whole‐body exercise at the same relative intensity (% V̇O_2max_ or Ẇ_peak_) or for either single‐joint or whole‐body exercise performed to exhaustion.

## 6 NEUROMUSCULAR FATIGABILITY IN NATIVE HIGHLANDERS

Differently from native lowlanders, who occasionally and temporarily migrate to high altitude, native highlanders, by virtue of their lineage and a lifetime in such an environment, present unique genotypic and phenotypic adaptations to live and perform with chronically low P_b_ and P_I_O_2_. Currently, successful inherent adaptations to high altitude are mainly recognized in three populations: Ethiopians, Tibetans and Andeans.^145^ Their patterns of adaptations, however, are markedly different. At altitudes ≥3500 m (P_I_O_2_ ≤ ~96 mm Hg), relative to lowlander natives at SL: (1) Andeans have greater [Hb] (resulting from similar plasma volume but greater haemoglobin mass) and lower S_a_O_2_; (2) Tibetans have similar [Hb] (due to greater plasma volume and haemoglobin mass) and lower S_a_O_2_; (3) Ethiopians have similar [Hb] and S_a_O_2_.^145^, ^146^, ^147^

Most of the studies on exercise performance of high‐altitude natives involve either Andeans or Tibetans, which means data are relatively scant in Ethiopians. Present day Andeans and Tibetans are descendants of people who first inhabited the Andean and Tibetan Plateaus ~11 000 and 25 000 years ago, respectively.^24^, ^148^ As such, despite the same external stressor at high altitude, ie, reduction in P_I_O_2_ of at least ~35% relative to SL, the two populations have adapted independently and differently. Despite similarities at the muscle ultrastructural level,^25^, ^30^ compared with native Andeans, native Tibetans typically have greater V̇_E_, lower mean pulmonary arterial pressure, lower [Hb] (because of greater plasma volume and lower haemoglobin mass), lower S_a_O_2_ and therefore lower C_a_O_2_, and greater capillary density.^22^, ^26^, ^145^, ^146^, ^149^ Such differences make it likely that neuromuscular performance and fatigability at high altitude will vary between the two populations. However, because there are no studies to compare neuromuscular fatigability between Tibetans and Andeans, and the only two studies to draw comparisons between lowlanders and native highlanders were conducted with Sherpa,^150^, ^151^ the Tibetan population will be the focus of this review. Of note, because Sherpa belong to an ethnic group that emigrated from Tibet ~500 years ago,^152^ Tibetans and Sherpa will be considered together, as Himalayans, as in previous reviews (eg, Ref. [27]). The following section will consider physiological adaptations as they relate to neuromuscular fatigability with exercise. For reviews that focus on physiological adaptations for life at high and extreme altitudes relative to lowlanders, we refer the reader to one of these seminal articles (eg, Refs [21, 22, 26, 27, 153]).

To investigate intrinsic fatigability of muscles of lowlanders and Sherpa at high altitude, we recently assessed force loss during a protocol of intermittent, electrically evoked knee extensor contractions, which minimized activation of the quadriceps via central pathways.^150^ Peak force declined less for Sherpa than lowlanders during the fatiguing task (Figure 6). Further, during the recovery period after the task, rapid force production was greater for Sherpa compared with lowlanders. Muscle oxygenation indices obtained with near infrared spectroscopy and estimated DO_2_ were not different between groups, which indicates that adaptations at the muscular level of Sherpa, independent of convective DO_2_, favour the preservation of muscle performance and repeated force production at high altitude.^150^ Sherpa, compared with lowlanders at high altitude, have less perturbation of phosphocreatine and inorganic phosphate levels, greater resting concentration of ATP and phosphocreatine (ie improved energetic reserve), and greater reliance on phosphocreatine hydrolysis.^28^, ^154^ Additionally, they have reduced glycolytic activation and anaerobic glycolytic enzymes, greater coupling between ATP supply and demand, decreased capacity for fatty oxidation, greater mitochondria coupling efficiency (conferring an overall greater efficiency of oxygen utilization), greater protection against oxidative stress and greater myoglobin content.^21^, ^23^, ^28^, ^154^, ^155^, ^156^ At the ultrastructural level of muscle, Sherpa, relative to lowlanders, have smaller mean fibre cross‐sectional area, greater capillary density (favouring greater O_2_ conductance), and lower mitochondrial density, but greater O_2_ consumption‐to‐mitochondria volume ratio.^157^ All these adaptations, besides contributing to the ‘lactate paradox’ for Sherpa at high altitude (ie, a lower‐than‐expected accumulation of blood lactate in a V̇O_2max_ test in native highlanders compared with lowlanders),^155^ would also make the muscles of Sherpa more resistant to peripheral fatigability. Specifically, these adaptations would allow lower accumulation of inorganic phosphate and lead to faster metabolite clearance and recovery, which would result in a lesser perturbation to the muscular milieu for Sherpa than lowlanders and confer a contractile advantage at high altitude.

The greater capacity for maintenance of neuromuscular homeostasis at high altitude in Sherpa relative to acclimatized lowlanders was recently noted during a sustained, voluntary isometric elbow flexion contraction at 25% MVC torque.^151^ Of note, due to the smaller stature and body mass of Sherpa than lowlanders, elbow flexor MVC torque was considerably lower (~37%) in the former group, which meant a lower absolute torque at the relative submaximal target.^151^ Despite similar supraspinal fatigability, biceps brachii motor‐evoked potentials, maximal M‐waves, and indices of cerebral oxygenation were less affected by the fatiguing task for Sherpa than lowlanders, indicating a lesser perturbation of homeostasis in the former group.

As muscles are the end point of the locomotor chain, these aforementioned differences for fatigability between lowlanders and Sherpa during single‐joint exercise are likely to greatly contribute to the superior performance at altitude for Sherpa during whole‐body activity. Notably, numerous studies have identified other advantageous physiological traits for performance at high altitude that Himalayans exhibit. Himalayans have greater V̇O_2max_ than native lowlanders acclimatized at high altitude,^158^, ^159^ as well as lower V̇_E_, lower pulmonary vascular resistance, a narrower alveolar‐to‐arterial P_a_O_2_ difference, a lesser decline in S_a_O_2_ and greater P_a_CO_2_ during exercise.^159^, ^160^, ^161^ For a review of these cardiovascular and respiratory adaptations, we encourage the reader to consult the following articles: Refs [21, 23, 26, 27, 38, 145, 153, 156, 162]. To view a graphical summary, see figure 2 in Ref. [27] or figure 1 in Ref. [23]. A tabular summary can be viewed in table 2 in Ref. [27].

From a performance perspective, differences between Himalayans and lowlanders have been reported for the economy of locomotion (ie, metabolic energy per unit distance). That is, when Himalayans and lowlanders cycled at the same work rate at high altitude, the former group exhibited lower V̇O_2_.^163^, ^164^ This disparity between populations has also been confirmed with walking and running at low altitude (1300 m).^165^ The greater economy of native highlanders compared with lowlanders seems likely to be advantageous for physical performance at high altitude, presumably yielding lower neuromuscular fatigability. However, the latter point is speculative, as neuromuscular fatigability (peripheral, central and supraspinal) with whole‐body exercise at high altitude has never been compared between native Himalayans and lowlanders. As such, experimental evidence is required.

The capacity of native Himalayans for superior performance at high altitude is exemplified by Sherpa porters, who can carry loads up to 200% of their body mass.^166^ When compared with lowlanders, for the same magnitude of carried load, Sherpa porters have a considerably lower cost of locomotion,^167^ greater mass‐specific metabolic power and locomotion efficiency.^168^ This superior performance is largely the result of biomechanically independent factors, namely respiratory, circulatory and muscular adaptations in native highlanders.^167^, ^168^ Such aspects should intuitively lead to lower fatigability in Sherpa porters compared with lowlanders for the same load‐carrying task; however, again, experimental evidence is needed to confirm these suppositions.

## 7 ADDITIONAL CONSIDERATIONS

The following factors are important to emphasize for interpretation of the findings presented in this review: how high altitude‐related hypoxia is induced (ie, real or simulated), the duration of acclimatization for lowlanders, the measures used to study peripheral fatigability, and the training status of lowlanders and native highlanders.

### 7.1 Inducing high altitude‐related hypoxia

For the study of AH, nearly all investigations have used simulated high altitude because it enables rapid delivery of a hypoxic stimulus, without giving enough time for adjustments to occur related to acclimatization. If participants travel to an elevation >3500 m, it typically takes hours to days for the ascent (depending on the means of transport), which means the study of AH would be biased by the initial adaptations that occur with acclimatization. Simulated AH may be achieved via normobaric or hypobaric hypoxia (ie, reducing F_I_O_2_ and P_b_, respectively; see Formula 2), with both leading to reduced P_I_O_2_. For this reason, it is not uncommon for researchers to compare data obtained via these two approaches; however, equivalence of the two conditions should not be assumed.^169^, ^170^, ^171^, ^172^ Hence, with simulated normobaric hypoxia being the most widely used paradigm to study AH, one must consider that these findings may differ from the results that would be obtained with either simulated hypobaric hypoxia or immediate ascent to high altitude.

In contrast to the study of AH, the investigation of neuromuscular fatigability with CH has relied almost exclusively on expeditions to high altitude. Whereas rarely used, simulated CH (eg, Operation Everest II)^72^ holds some advantages over field work in that it allows factors such as dietary requirements or daily exercise to be controlled, thereby better isolating the effect of hypoxia from others factors inherent to an expedition.

### 7.2 Duration of acclimatization for lowlanders

In the present review, we considered both real and simulated altitudes between 3500 and 6250 m, and short‐ to medium‐term acclimatization (ie, ~7‐28 days) as CH. However, even when excluding elevations below or above this range as well as very brief or prolonged acclimatization periods, the hypoxic stimulus varies markedly among studies. When considered in conjunction with the inherent between‐participant variability and the small sample sizes for these studies, there are many research questions still to be answered and findings to be confirmed.

### 7.3 Measures to study peripheral fatigability

Irrespective of environmental conditions, to accurately depict fatigue‐related impairments in muscle contractility, force responses should be collected via supramaximal stimulation across a broad range of frequencies. Although a full force–frequency relationship would be ideal, the procedure is time consuming, which means that the muscle state could differ among responses as the muscle recovers from the fatiguing protocol. Further, depending on the muscle group examined, stimulation trains of sufficient duration (usually 1 s) to elicit a plateau of force for each tetanic contraction can be prohibitively uncomfortable or yield unreliable force responses. As such, force responses to single or paired high‐frequency stimuli (twitches and doublets, respectively) are often used. Whereas this is a practical compromise, the lower number of stimuli can lead to an overestimation of peripheral fatigability, especially at high stimulation frequencies^173^ and acutely post‐exercise.^174^ This makes the decline of twitch or doublet force a poor measure of peripheral fatigability. When a full force–frequency relationship is not feasible, it is advised that responses be collected at both low‐ and high‐frequencies of stimulation^84^ as well as the frequency that approximates the motor unit discharge rates expected during the voluntary contractions of the fatiguing task. The majority of studies to investigate peripheral fatigability with AH and CH used only twitch or doublet force, which means there is still much to learn about intrinsic fatigability of muscle fibres under these conditions.

### 7.4 Training status of lowlanders and native highlanders

To draw appropriate conclusions about the influence of heritable adaptations and a lifetime of high‐altitude exposure on neuromuscular fatigability (or any physiological measure) in such an environment, ideally participants of each group should have an equivalent long‐term training status. Although training status was not quantified in any of the studies we cite, the lowlanders who participate in such expeditions tend to be active and fit individuals, so we believe they are likely to represent a comparably trained group to native highlanders. Nevertheless, it would be valuable for future studies to confirm this with some measure of long‐term physical activity. Given the logistical challenges associated with equipping highlanders with wearable technology (eg, an accelerometer) prior to an expedition, a questionnaire (eg, Global Physical Activity Questionnaire)^175^ is likely the most feasible option. However, appropriate translation into the native language of the highlander population would be very important for a meaningful comparison with lowlanders (although differences in the interpretation of questions may still persist due to cultural reasons).

## 8 FUTURE DIRECTIONS

Despite a growing body of knowledge regarding the effects of acute and chronic exposure to high altitude on neuromuscular function and fatigability, several issues remain to be addressed. For example, eccentric versus concentric contractions, which differ for neural contributions at the spinal and supraspinal levels,^176^ could be used in fatiguing tasks to determine whether the effect of AH and CH on neuromuscular fatigability is specific to contraction type. Furthermore, given the apparent preservation of motoneurone excitability during fatiguing exercise with CH,^19^ studies should examine activity of single motor units (eg, Ref. [177]) to determine to what extent AH and CH affect motoneuronal output, and how this relates to task performance (eg, force steadiness). Although expeditions to high altitude have provided mechanistic insight into physiological adaptations to sustained severe hypoxia (ie, acclimatization), future expeditions should endeavour to include the study of adaptations that occur following the return to SL (ie, de‐acclimatization). Field expeditions should also extend our recent work with single‐joint exercise,^150^, ^151^ and compare peripheral, central and supraspinal fatigability between lowlanders and native highlanders with whole‐body exercise. Finally, a comparison of fatigability among the three different ethnic groups at high altitude (ie, Ethiopians, Himalayans and Andeans) would provide invaluable information regarding the processes by which humans have evolved to their environment, and the functional consequences from a neuromuscular perspective.

## 9 CONCLUSIONS

In the current review, we summarized findings of the influence of high altitude‐related hypoxia (ie, 3500‐6250 m, P_I_O_2_ between ~64.3% and 43.6% of that at SL) on neuromuscular fatigability during single‐joint or whole‐body exercise in the contexts of acute exposure, short‐ to medium‐term (~7‐28 days) acclimatization, as well as heritable adaptations and lifetime residence. It is clear from the results that peripheral and supraspinal fatigability worsen with acute exposure to high altitude. However, if the exercise is conducted to exhaustion, peripheral fatigability will be lower, whereas supraspinal fatigability will be greater. With acclimatization, both measures are restored to values at SL for single‐joint exercise. In contrast, with whole‐body exercise, only supraspinal fatigability is restored to the sea‐level standard, whereas peripheral fatigability is not improved relative to acute high‐altitude exposure. Compared with acclimatized lowlanders, native Himalayan highlanders (Tibetans and Sherpa) present lower peripheral fatigability and greater maintenance of neuromuscular homoeostasis during single‐joint exercise. Along with known differences between Himalayans and lowlanders for the cardiovascular and respiratory systems, the implied neuromuscular adaptations for highlanders would presumably lead to lower neuromuscular fatigability compared with lowlanders for a whole‐body exercise at high altitude; however, this has yet to be addressed experimentally.

## CONFLICT OF INTEREST

There are no competing interests to declare.

## AUTHOR CONTRIBUTIONS

All authors contributed to the conception of the present review. LR and SH collected information from individual studies and drafted the manuscript. All authors revised the manuscript critically for important intellectual content. All authors have read and approved the final submission.
